# Combination of In Situ Diffraction Experiments and Acoustic Emission Testing to Understand Compression Behavior of Mg-Gd Alloys

**DOI:** 10.3390/ma17225654

**Published:** 2024-11-20

**Authors:** Gerardo Garces, Bryan W. Chavez, Pablo Pérez, Judit Medina, Kristian Mathis, Rafael Barea, Andreas Stark, Norbert Schell, Paloma Adeva

**Affiliations:** 1 Department of Physical Metallurgy, National Centre for Metallurgical Research CENIM-CSIC, Avda, Gregorio del Amo 8, 28040 Madrid, Spain; zubiaur@cenim.csic.es (P.P.); judit.medina@cenim.csic.es (J.M.); adeva@cenim.csic.es (P.A.); 2 Escuela Politécnica Superior y de Arquitectura, Universidad Nebrija, C. Sta. Cruz de Marcenado, 27, 28015 Madrid, Spainrbarea@nebrija.es (R.B.); 3 Faculty of Mathematics and Physics, Charles University, Ke Karlovu 5, 121 16 Prague, Czech Republic; kristian.mathis@matfyz.cuni.cz; 4 Institute of Nuclear Physics of the CAS, Husinec—Řež, čp. 130, 250 68 Řež, Czech Republic; 5 Institute of Materials Physics, Helmholtz-Zentrum Hereon, Max-Planck-Str. 1, 21502 Geesthacht, Germany; andreas.stark@hereon.de (A.S.); norbert.schell@hzg.de (N.S.)

**Keywords:** magnesium alloys, mechanical properties, synchrotron radiation diffraction

## Abstract

The compressive deformation of the extruded binary Mg-Gd with gadolinium in solid solution has been studied in situ by combining synchrotron diffraction and acoustic emission techniques during compression tests. These two techniques are useful in investigating the evolution of twinning in all its stages. The extruded bars develop a fiber texture with the basal plane parallel to the extrusion direction. Moreover, the quenching of the magnesium bars immediately after the extrusion process ensured the production of the solid solution of gadolinium in the magnesium matrix. The solid solution of gadolinium solute atoms is the main strengthening mechanism of alloys and has a strong influence in plastic deformation. Tensile twinning controls the macroscopic yielding under compressive modes, although the activation of basal and non-basal dislocation systems has been also detected by in situ techniques. The presence of gadolinium atoms in solid solution tends to inhibit tensile twinning and, therefore, the twin volume fraction decreases with the increase in the gadolinium content. The compressive work hardening curve shows a maximum peak at intermediate plastic strain which is related to the interaction of dislocations within twins. The maximum value and the position of the peak decreases with the increase in the gadolinium content.

## 1. Introduction

The addition of rare-earth (RE) to magnesium alloys has a strong influence on the corrosion behavior, formability, ignition resistance, and mechanical properties of magnesium alloys [1,2,3,4,5,6,7,8,9,10,11,12,13,14,15]. In terms of corrosion, several reasons have been described for this improvement. RE elements can decrease the effect of impurities by trapping them in intermetallic compounds [16]. These new intermetallic phases have lower potentials and reduce significantly micro-galvanic couples [7,17]. The improvement of formability due to the addition of RE is directly connected to the decrease in fiber texture developed in wrought alloys. The texture modification and RE texture formation were attributed to several different mechanisms such a solute segregation at grain boundaries, dynamic strain aging, particle stimulating nucleation at the intermetallic phases, solute drag, preferred growth of oriented nuclei, and the decrease in the stacking fault energy [18,19,20,21,22,23]. REs elements behave as a reactive element effect for enhancing the ignition resistance of Mg alloys in two ways [24,25]. In one way, REs react with oxygen to form a dense oxide film that delays the diffusion of Mg^2+^ cations and oxygen ions and therefore limit the growth of MgO. In the second way, REs with high solid solubility, such as Y or Gd, condense into cracks in the MgO layer and react preferentially with oxygen to compensate for the diffusion paths formed in the oxide layer.

The improvement of mechanical properties occurs mainly in two ways. The first way is through the precipitation of thermally stable second-phase particles that increase their mechanical strength and improve the creep resistance. Binary or ternary Mg-REs systems present precipitation sequences that involve the development of coherent, semi-coherent, or incoherent precipitates that induce a maximum of hardness after an aging treatment [26]. The formation of plate-shape precipitates located at prismatic planes are an effective barrier to the difficult slip of <a> dislocations along the basal plane [27]. At high temperature, the stable intermetallic particles have a higher Young’s modulus compared to magnesium, which can bear an additional load transferred from the soft magnesium matrix.

The second way occurs because REs have a great reinforcing effect as they increase the mechanical strength when RE atoms are in solid solution [28,29]. Thus, the yield strength follows a potential law with c^n^, where c is the content of Gd, and *n*  =  1/2 or 2/3 [30]. Therefore, the higher the RE content in solid solution, the higher the reinforcing solute effect. Yttrium and gadolinium exhibit a high solubility in magnesium (around 20 wt.%). Among both rare earth elements, gadolinium is more interesting as an alloying element due to its low tendency to form oxide inclusions during alloy processing [31]. Recently, Gu et al. [32] have reported that the solid solution strengthening effect of extruded Mg–Gd alloys mainly occurs in the composition range of 1–4 wt.%. However, Xu et al. [30] reported solid solution strengthening also at higher solute contents (around 10 wt.%).

Gadolinium atoms interact strongly with dislocations or segregate at grain and twin boundaries [33,34,35,36,37,38,39,40]. This effect is significant in extruded samples under compression where tensile twinning can be activated. The presence of gadolinium in solid solution enhances the activation of the non-basal slip system and tends to substantially reduce the activation of tensile twinning [41,42]. This fact is directly manifested in the evolution of work hardening as a function of the gadolinium content [41,42,43]. The work hardening curve during compressive plastic deformation exhibits different stages. After the initial minimum, corresponding to macroscopic yielding of the extruded alloy, the work hardening increases with the increase in compressive strain up to a maximum. Following the same nomenclature of Imandoust et al. [43], this stage is defined as region II. The maximum value of the peak is shifted to a lower compressive strain that decreases with the rise in gadolinium content. However, the evolution of the maximum peak value of the work hardening as a function of gadolinium content follows two opposite behaviors. In one behavior, Kula et al. [41] and Yang et al. [42] reported that the height of the work hardening peak decreases with the increase in the gadolinium content. Moreover, the full width half maximum (FWHM) of this peak increases with the increase in the gadolinium content. In the other behavior, the evolution of work hardening reported by Imandoust et al. [43] shows the opposite. The interaction of {10$\bar{1}$2} twin boundaries with basal <a> and 〈c + a〉 dislocations cause the rise of the work hardening rate in Region II [44,45]. After the peak, the work hardening decreases again (Region III) due to the activation of dislocations slip (principally basal slip) within twins.

In accordance with what was stated above, the objective of this work is to analyze the mechanisms of plasticity and work hardening during in situ compression tests in two binary Mg-Gd alloys with gadolinium in solid solution, combining techniques of synchrotron radiation diffraction (SRD) and acoustic emission (AE). Furthermore, the microstructure of the samples deformed under different stresses will be studied by electron backscattered diffraction (EBSD) and transmission electron microscopy (TEM).

## 2. Materials and Methods

### 2.1. Materials

Two binary Mg-Gd alloys with 1 and 6 wt.% (0.22 and 1 at.%) were prepared by melting and casting using an electric furnace with adequate proportions of high purity Mg and a Mg-30 wt.%Gd master alloy. The cast alloys were solubilized at 550 °C and buried in MgO to minimize oxidation. After 24 h, the cast billets were quenched and machined obtaining cylinders of 41 mm in diameter and 150 mm in length. Finally, cylinders were extruded at 450 °C at an extrusion rate of 0.5 mm·s^−1^ with an extrusion ratio 25:1. During extrusion, the bars were quenched immediately in order to retain the gadolinium atoms in solid solution. For comparison purposes, pure magnesium was also extruded at the same condition as the binary alloys. The three materials were designated G0, G1, and G6 for the pure magnesium, Mg-1%Gd and M-6%Gd, respectively.

### 2.2. Microstructural and Mechanical Characterization

Microstructural characterization of the alloys in extruded condition and after plastic deformation was performed by scanning (SEM) and transmission electron (TEM) microscopy. The samples for SEM were prepared by mechanical polishing, finishing with an etching solution of 5 mL acetic acid, 20 mL water and 25 mL picric acid in methanol. Specimens for TEM after deformation were prepared by electrolytic polishing using a reactive mixture of 5.3 g lithium chloride, 11.2 g magnesium perchlorate, 500 mL methanol, and 100 mL butoxy-ethanol at −50 °C.

The crystallographic texture of the alloys was evaluated using electron backscattered diffraction (EBSD) technique. The specimens for EBSD were etched using a solution of 7 mL acetic acid, 3 mL of nitric acid, 30 mL of ethanol, and 10 mL of water. EBSD measurements were carried out in a direction perpendicular to the extrusion direction and data were recorded and analyzed using Channel 5 software. The macroscopic texture was evaluated using a Rietveld texture analysis in MAUD [46]. The 2D detector image was converted to a set of diffraction patterns using the Image J plugin (software 2.9.0) in the software [47,48]. The best fit for α-Mg (P63/mmc) diffraction peaks was found using the E-WIMV algorithm with 5° resolution. A fiber texture was assumed during the calculation. This procedure was repeated during the compression test obtaining the evolution of the texture as a function of the compressive strain. From the (0002) pole figure, the volume fraction of twins can be calculated following the procedure reported by Agnew et al. [49] given by:

(1) $$f_{tw}=\int_{\varphi =0}^{\varphi \approx 57{}^{\circ}}\left[I\left(\varphi \right)-I_{0}\right]\;sin\varphi \;d\varphi$$

where φ is the angle of tilt between the (0002) poles and the compression axis and I(φ) is the peak intensity for the given angle φ.

The compression tests were carried out in a universal tensile rig at initial strain rates of 10^−4^ s^−1^. Cylindrical samples were mechanized along the extrusion direction with dimensions of 5 mm diameter and 10 mm length. A synthetic grease was used to minimize the friction between surfaces of the specimen and the compression plate.

### 2.3. In Situ Synchrotron Radiation Diffraction

Synchrotron radiation diffraction (SRD) experiments during compression tests were performed on the P07 beamline of PETRA III at the Deutsches Elektronen-Synchrotron (DESY). In situ tensile compressive tests were carried out using a DIL805A/D dilatometer from TA Instruments at a strain rate of 10^−3^ s^−1^. Compressive samples were machined from the bar with the round section along the extrusion direction with special dimensions defined by the dilatometer. Samples for the compression tests were cylinders of 5 mm in diameter and 10 mm in length machined along the extrusion direction. The gauge volume was defined by 0.8 × 0.8 × 5 mm^3^ (beam section × cylinder diameter). The diffraction patterns were recorded using an exposure time of 0.5 s by a Perkin-Elmer XRD 1621 flat panel detector with an array of 2048^2^ pixels^2^ and an effective pixel size of 200 × 200 µm^2^. The wavelength was 0.0124 nm. LaB6 was used as reference to calibrate the system. The detector-to-sample distance was 1621 mm. Conventional 2θ diffraction profiles were obtained by azimuthal integration of the Debye–Scherrer rings in the axial and radial direction around ±7.5°. The fitting of the diffraction peaks was carried out with the FIT2D software [50] using a Gaussian function. The lattice strain for each orientation can be calculated by the relative shift in the position of the diffraction peak defined by:

(2) $$\varepsilon_{hkl}=\frac{d_{hkl}-d_{0,hkl}}{d_{0,hkl}}$$

where d_hkl_ and d_0,hkl_ are the planar spacing of the hkl plane in the stressed and stress-free crystal. The value of the internal deformations for σ_ap_ = 0 MPa is 0 µstrains under the assumption that the extruded material does not exhibit residual stresses (RS). For materials with RS, other methods such as using powders or machining a free RS comb should be used.

The lattice spacing and the diffraction angle θ are related through Bragg’s law:

(3) $$d_{hkl}=\frac{\lambda }{{2sin\theta }_{hkl}}$$

### 2.4. In Situ Acoustic Emission

The AE measurement was carried out during compressive tests at the same deformation conditions used in the in situ SRD experiments. PCI-2 acquisition board (Mistras Corp., Princeton, NJ, USA) was used for recording of the AE signal. The raw acoustic emission was in a data streaming regime where the data were recorded continuously with 18-bit amplitude resolution and 1 MHz sampling rate. A physical acoustic S80 micro sensor and a 2–4–6 preamplifier were used with a gain set to 40 dB. The piezoelectric AE sensor was mounted directly onto the sample using a cloth pin.

Since multiple AE sources are simultaneously active during Mg alloy straining, and there is a huge AE signal during the entire test, traditional hit-based processing, where a threshold and a hit definition time are used to divide the signal into separate AE events (see [51] for details), fails.

Therefore, we applied the adaptive sequential k-means (ASK) cluster analysis [52]. In this method, the frequency characteristics of the signal is used for identifying the dominant deformation mechanisms within in a given time period (defined as frames). Therefore, the continuously recorded data are sectioned into consecutive analytical frames. In our case, one frame contained 2048 samples, which corresponds to a 2 ms wide analytical window. For each frame, the power spectral density (PSD) function was calculated. Due to the random character of the AE, there was a significant scatter in the PSDs. Thus, discrimination between signals stemming from different sources can be performed only on a statistical basis. Kullback–Leibler distance was used as a measure of the similarity of the shapes of normalized PSDs. The k-means procedure was used for clustering. Since the recording of AE was launched a few seconds before starting the deformation test, the first cluster naturally belongs to background noise, and all other clusters are related with respect to this noise cluster. Once the signals in all frames are divided into individual clusters, a dominant AE source mechanism is associated with each cluster based on the time of appearance and mutual distributions of physical parameters (amplitude, energy, frequency range) of the cluster elements. Finally, the relative source activity can be plotted, which indicates which mechanism is dominant at a given stage of the straining. However, this does not mean that other mechanisms cannot be concurrently active.

## 3. Results

### 3.1. Microstructure

**Figure 1:**
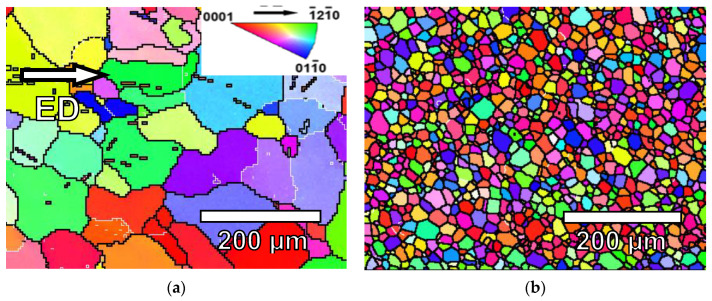
Orientation image mappings of extruded (**a**) magnesium and (**b**) G6 alloy along the extrusion direction.

Figure 1 shows the grain structures along the extrusion direction of the G0 and G6 samples. The grain structure is fully recrystallized and the addition of gadolinium significantly reduces the grain size. No second phases were observed. Previous studies concluded that gadolinium atoms are in solid solution [53]. During extrusion, grains orient their basal planes parallel to the extrusion direction.

### 3.2. Compressive Behavior

**Figure 2:**
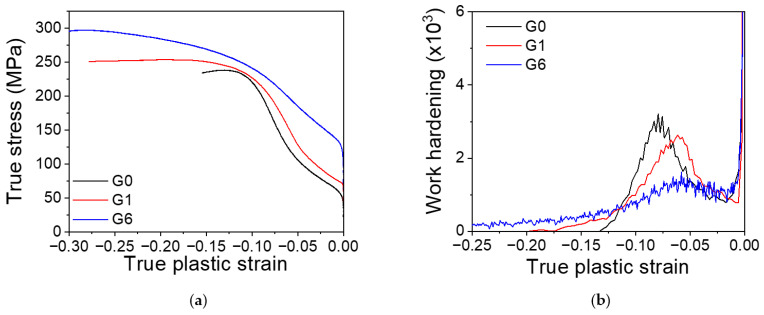
(**a**) Compressive true stress–true plastic strain curves of G0, G1, and G6. (**b**) Work hardening as a function of the true plastic strain of G0, G1, and G6.

The compression tests of the three extruded materials G0, G1 and G6 are shown in Figure 2a. Moreover, the work hardening exponents obtained from these curves are also plotted in Figure 2b. Compression curves exhibit the typical sigmoidal shape due to the activation of tensile twinning. Extruded pure magnesium (G0) yields at a stress close to 50 MPa. At the beginning of the plastic deformation, the work hardening reached a minimum around 2% of compressive plastic strain. At 4% of compressive plastic strain, the stress begins to increase rapidly, and the work hardening presents a maximum at a compressive strain of 8%. From this deformation, the hardening decreases continuously. At 12% plastic deformation, the compression curve flattens; therefore, work hardening is zero. Finally, the compression sample fractures at 15% of compressive plastic strain.

The G1 alloy shows similar compressive behavior as the pure magnesium. However, the yield stress is slightly higher (80 MPa) and the maximum work hardening at intermediate plastic strains is shifted to a lower macroscopic compressive plastic strain.

The compressive curve of the extruded G6 alloy, which exhibits the highest gadolinium content in solid solution, is different. Yield stress increases significantly compared with the G0 and G1 alloys. The shape of the curve also has a sigmoidal character but not as pronounced as with the other two materials. Moreover, the evolution of work hardening as a function of the compressive plastic strain showed a maximum at 5% of strain as the extruded G1 alloy, but the work hardening value is much lower. After the maximum, the work hardening decreases continuously without the presence of a stress plateau, characteristic for the G0 and G1 samples.

### 3.3. In Situ Synchrotron Radiation Diffraction During Compression Tests

**Figure 3:**
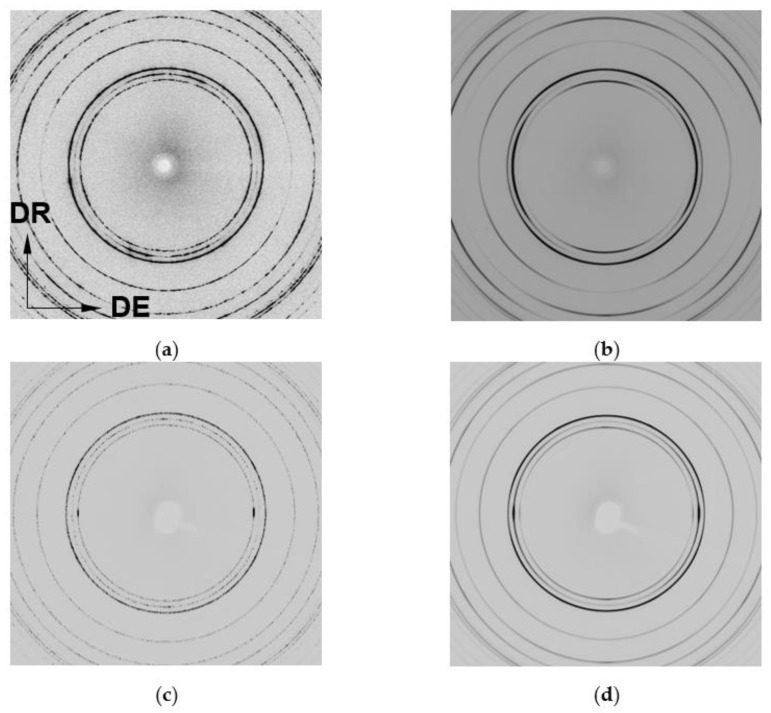
Synchrotron radiation diffraction patterns recorded on the 2D flat panel detector before and after the compressive test. G0 at (**a**) 0 and (**b**) 11% of strain and G6 at (**c**) 0 and (**d**) 8% of strain. (Axial and radial directions are marked in (**a**)).

Figure 3 shows the Debye–Scherrer patterns obtained in the 2D detector for the extruded pure magnesium and extruded G6 alloy before starting the compression test (ε = 0%) and at the end of the test, before starting the unloading. Before starting the test, the diffraction rings show a large number of spots inside for the pure magnesium, which can be ascribed to coarse grains observed in Figure 1a. As plastic deformation increases, the rings tend to be continuous due to the formation of a deformed structure within the original grains. The intensity changes with respect to the initial states are caused by the evolution of the texture during compression.

**Figure 4:**
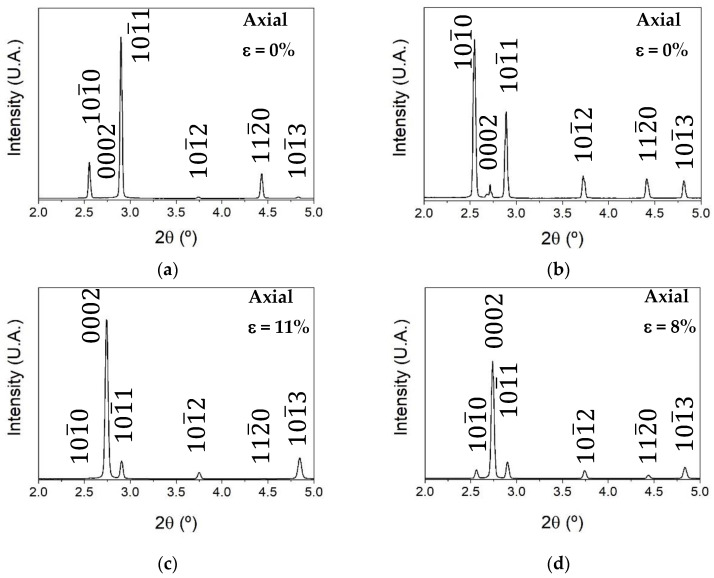
Axial diffraction patterns as a function of 2θ obtained by integration of the Debye–Scherrer rings. G0 at (**a**) 0 and (**c**) 11% of strain and G6 at (**b**) 0 and (**d**) 8% of strain.

The Debye–Scherrer patterns are integrated along axial and radial directions between an angle of ±7.5° (15° in total) to obtain the typical 2θ diffraction patterns. Figure 4a–d shows diffraction patterns as a function of 2θ for the G0 and G6 samples at ε = 0% and ε = 11% or 8% in the axial direction. Due to the texture of the extruded bars, where grains are preferably oriented with the basal planes parallel to the extrusion direction, the most intense peak in the axial direction corresponds to the {10$\bar{1}$0} diffraction peak. This phenomenon is reversed for radial direction where the most intense diffraction peak corresponds to {0002}. After the compression test, the intensity of the {0002} diffraction peak in the axial direction has increased at expense of the {10$\bar{1}$0} diffraction peak.

**Figure 5:**
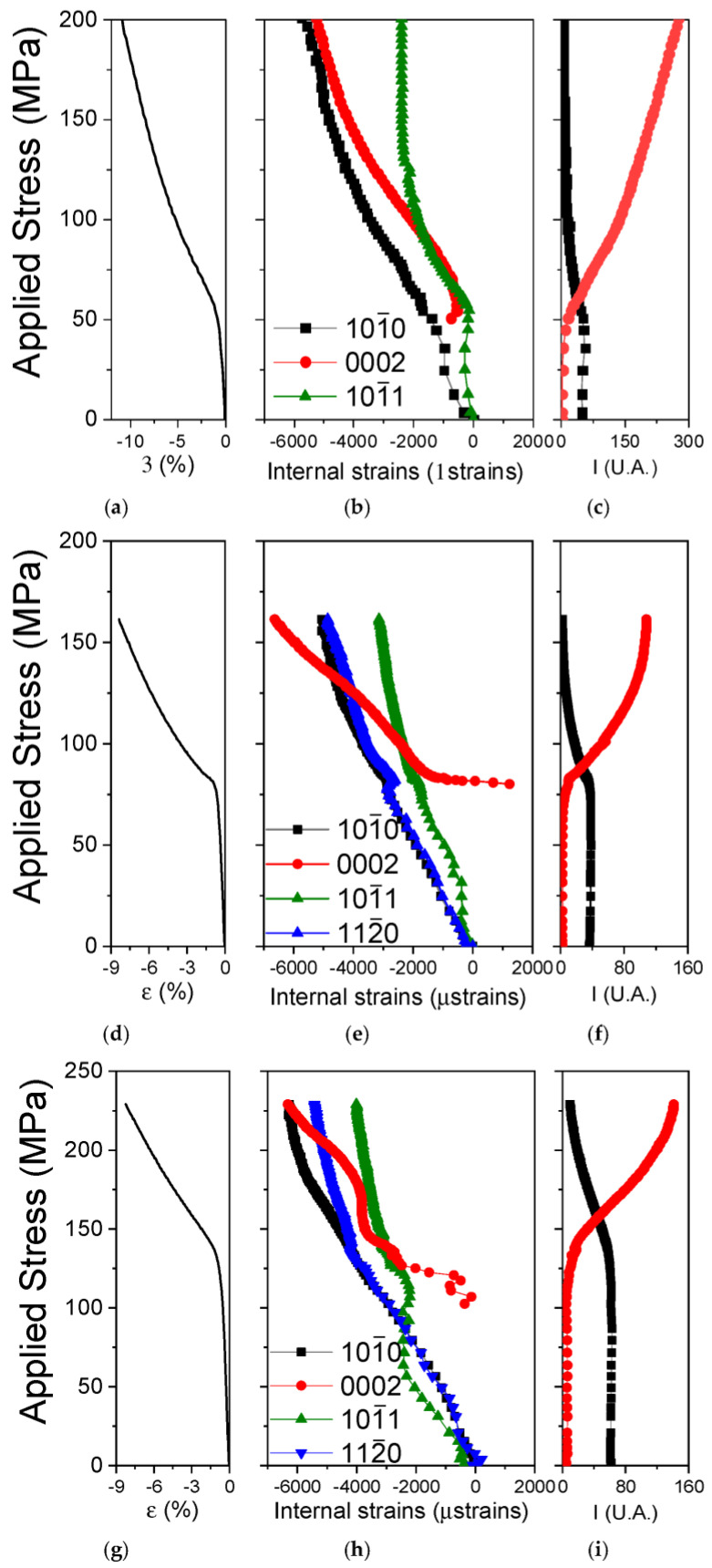
Compressive macroscopic stress-strain curve of the (**a**) G0, (**d**) G1, and (**g**) G6 alloys. Axial lattice strains as a function of the applied stress during an in situ compression test at room temperature for the (**b**) G0, (**e**) G1, and (**h**) G6 alloys. Evolution of the integrated intensity in the axial direction of the {10$\bar{1}$0} and (0002) diffracted peaks during compression for the (**c**) G0, (**f**) G1, and (**i**) G6 alloys.

The diffraction peaks from 2θ patterns are fitted individually, obtaining the value of the diffraction angle θ and their intensities. The elastic deformation has been calculated using Equation (2). Figure 5b,e,h shows the evolution of the lattice strains for the particular samples as a function of the applied stress for the diffraction peaks {10$\bar{1}$0}, {0002} and {10$\bar{1}$1} of the magnesium phase in the axial direction. The corresponding compression curves, which are similar to that in Figure 2, are shown in Figure 5a,d,g. Furthermore, the evolution of the intensities of the {10$\bar{1}$0} and {0002} diffraction peaks also in the axial direction are displayed to identify the beginning and evolution of the twinning process (Figure 5c,f,i).

The macroscopic elastic regime for G0 sample is below 49.5 MPa, as is shown in Figure 5a. The lattice strain corresponding to the grains oriented with the {10$\bar{1}$0} plane perpendicular to the compression axis shows linear elastic dependence with a slope close to 40 GPa. The lattice strain for the {10$\bar{1}$1} diffraction peak quickly loses its linearity at a stress level below 5 MPa. This peak provides information about the activation of the basal slip. The critical shear stress for the activation of this system in magnesium has been estimated as 0.5 MPa [54,55,56]. Thus, such a microyielding [57,58,59] observed here is comprehensible. Nevertheless, due to the low volume fraction of these grains, the macroscopic yield has to be controlled by another mechanism. After microyielding, the slope of the {10$\bar{1}$1} lattice strain increases and a value of 200 µstrain is reached at macroscopic yield stress. Above this stress level, the lattice strains increase linearly again up to an applied stress value of 100 MPa where the slope again increases progressively until reaching a value of 2300 µstrains.

The evolution of the lattice strains on the {10$\bar{1}$0} planes above the macroscopic yield is linear up to an applied stress of 150 MPa. At this stress, the slope increases, which can be ascribed to the activation of non-basal plastic deformation systems.

Due to the low intensity, the peak positions for the (0002) could not fit reliably in the macroscopic elastic regime. Therefore, the lattice strain values for (0002) were not determined below the macroscopic yield stress. In the plastic region, the lattice strain behaves similarly to that on {10$\bar{1}$0} planes. The integrated intensity of the {0002} peak increases progressively at the expense of the {10$\bar{1}$0} diffraction peak (Figure 5c). This effect is related directly to the activation of the tension twinning system {10$\bar{1}$2}〈10$\bar{1}$1〉. The basal plane rotates 86° with respect to the initial orientation of the grain. For this reason, the evolution of the elastic internal strains of the {0002} diffraction peak, above yield stress, is connected to twin formation and growth, which control the magnesium yielding.

Figure 5e shows the evolution of the lattice strains as a function of the applied stress for the diffraction peaks {10$\bar{1}$0}, {0002}, {10$\bar{1}$1} and {11$\bar{2}$0} of the magnesium phase, in the axial direction, for the extruded G1 alloy. The compression curve obtained during the in situ test (Figure 5d) and the evolution of the intensities of the {10$\bar{1}$0} and {0002} diffraction peaks (Figure 5f) are also plotted.

The shape of the evolution of the elastic deformations of the peaks studied is similar to those obtained for pure magnesium (G0). The yield stress of the extruded G1 alloy is 80 MPa. Below this stress, the curve shows purely elastic behavior. The lattice strains corresponding to primary and secondary prismatic planes ({10$\bar{1}$0} and {11$\bar{2}$0}) show a linear relationship with the applied stress in the elastic regime. However, grains oriented with {10$\bar{1}$1} planes perpendicular to the compression axis show a microyielding around 12 MPa. Although, as extruded pure magnesium, the volume fraction of these grains is small and they do not control the onset of macroscopic yielding of the extruded alloy.

Above yield stress, the evolution of the lattice strains on {10$\bar{1}$0} planes exhibit the same linear behavior as in the elastic regime up to 125 MPa. Above this stress, the slope increases and follows an asymptotic behavior towards 5000 µstrains. The evolution of the lattice strains on {11$\bar{2}$0} is similar to that on {10$\bar{1}$0} planes. Both lattice strains follow the same slope in the elastic regime with a value of 41 GPa. The evolution of the lattice strain on {10$\bar{1}$1} planes from the microyielding to 30 MPa does not change. Above this stress level, the lattice strain evolution copies the course of lattice strains on {11$\bar{2}$0} and {10$\bar{1}$0} planes.

The lattice strains corresponding to the (0002) diffraction peak (twins) can be again evaluated only above the macroscopic yield stress. It is important to mention that initial values obtained after yield stress are positive (tensile stress). Although, the sign of lattice strain values changes quickly and becomes negative (compressive stress). From 83 MPa, the lattice strains increase continuously with a small slope, which indicates softening caused by twinning. Accordingly, the intensity of the (0002) diffraction peak also increases (Figure 5f), which is connected to the twin growth.

Finally, Figure 5h shows the evolution of the lattice strains as a function of the applied stress for the {10$\bar{1}$0}, {0002}, {10$\bar{1}$1}, and {11$\bar{2}$0} planes in the axial direction for the extruded G6 alloy. The compression curve obtained during the in situ test (Figure 5g) and the evolution of the intensities of the diffraction peaks {10$\bar{1}$0} and {0002} (Figure 5i) are also plotted.

The general behavior of lattice strains on {10$\bar{1}$0}, {10$\bar{1}$1}, and {11$\bar{2}$0} planes is similar to the G1 alloy. Although, the applied stress values for the transition between the different regions, explained in detail above, increase due to its superior mechanical strength.

As in the two previous alloys, lattice strain on the (0002) plane is directly connected to twinning. However, its evolution is significantly different with respect to G0 and G1. The lattice strain curve exhibits a sigmoidal behavior. In its intermediate zone (between 2 and 4% of plastic deformation), the evolution of internal deformations has a constant value of −3800 µstrains. It is interesting to point out that this behavior can be perceived also in the extruded G1 alloy between 83 and 100 MPa. At higher applied stress, the internal deformation curve once again recovers the same slope observed above the yield stress.

**Figure 6:**
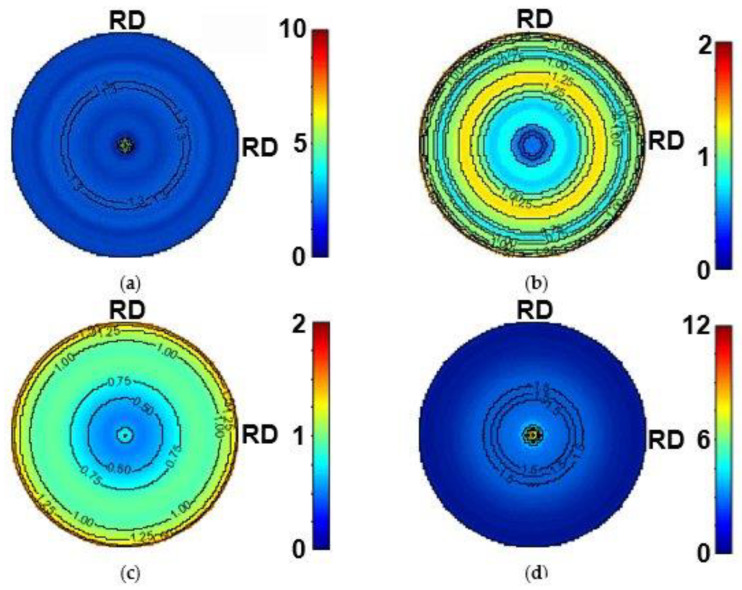

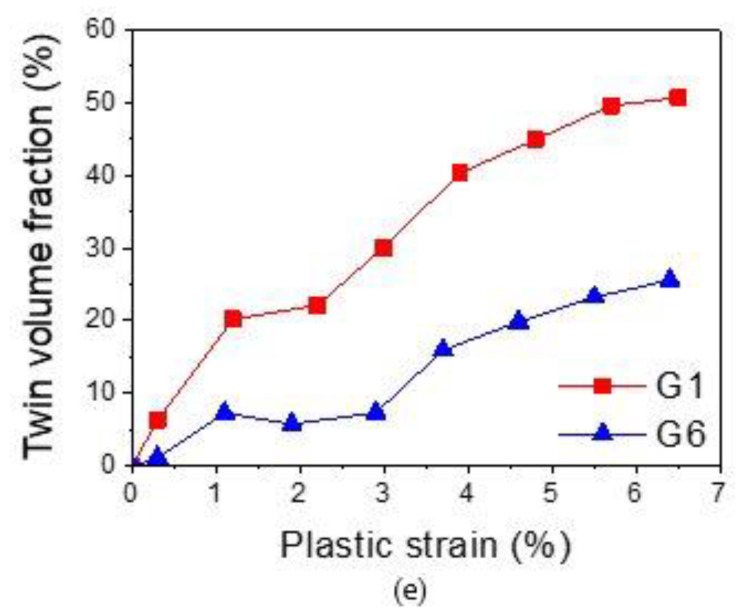
The {10$\bar{1}$0} and (0002) pole figures of extruded G6 alloys: (**a**,**b**) before and (**c**,**d**) after compression test in the synchrotron beamline. (**e**) Evolution of twin volume fraction as a function of the compressive plastic strain for extruded G1 and G6 alloys.

From the Debye–Scherrer 2D patterns, pole figures can be calculated along the compression test for the extruded G1 and G6 alloys. As an example, Figure 6a–d shows the {10$\bar{1}$0} and {0002} pole figures of the extruded G6 alloy before and after the compression test. The textures of the sample change completely with respect to the initial texture fiber with the basal plane parallel to the extrusion direction. During the compression test, the grains rotate their basal planes 90° and they are oriented perpendicular to the compression axis. These pole figures agree with the evolution of the intensity of the {10$\bar{1}$0} and {0002} diffraction peaks in the axial direction, shown in Figure 5. Using the {0002} pole figure, the twin volume fraction in the magnesium matrix has been calculated using Equation (1). Figure 6e shows the evolution of the twin volume as a function of the plastic compressive strain for the two binary Mg-Gd alloys. The twin volume fraction in both alloys increases with the increasing plastic strain. Comparing both alloys, the twin volume fraction decreases with the increasing gadolinium content. Therefore, it can be concluded that gadolinium atoms in solid solution hinder the nucleation or growth of twins.

### 3.4. In Situ Acoustic Emission During Compressive During Compression Tests

**Figure 7:**
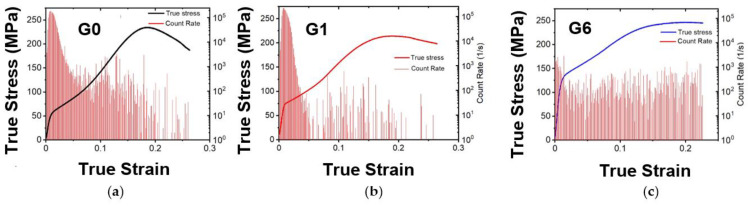
Evolution of the AE count rates and the corresponding deformation curves for (**a**) G0, (**b**) G1, and (**c**) G6 samples.

The evolution of AE count rate during compression test for three studied materials are shown in Figure 7. The characteristic S-shape of the compression curve indicates a massive twinning nucleation in the vicinity of the yield point. Accordingly, the AE activity is the largest in this stage of the deformation for all samples. Further, there is a significant AE activity already in the elastic regime [60]. Such a behavior is often observed in magnesium alloys and it clearly demonstrates the extremely sensitivity of the AE method to microplastic events. It is obvious from Figure 7 that the peak value of AE count rate decreasing with the increase in the gadolinium content.

As is mentioned in the experimental section, the ASK procedure [52,61] was used for identifying the dominant deformation mechanisms in particular parts of the straining.

**Figure 8:**
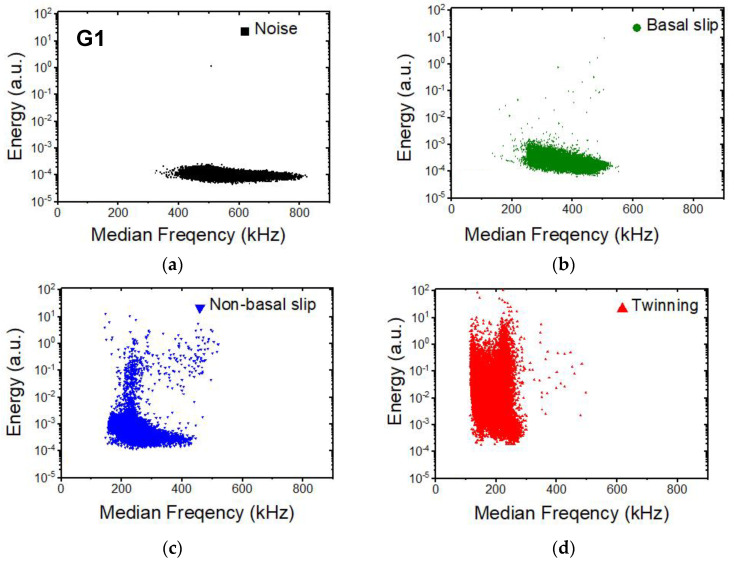
Distribution of AE clusters in energy–median frequency space using the ASK clustering method for the G1 sample. The clusters, represented by different colors, are assigned to particular source mechanisms, based on their characteristic features (energy, frequency distribution, etc.) ((**a**) Noise, (**b**) basal slip, (**c**) non-basal slip, and (**d**) twinning). The distribution of the clusters for the G0 and G6 samples looks similar, therefore it is not presented here.

**Figure 9:**
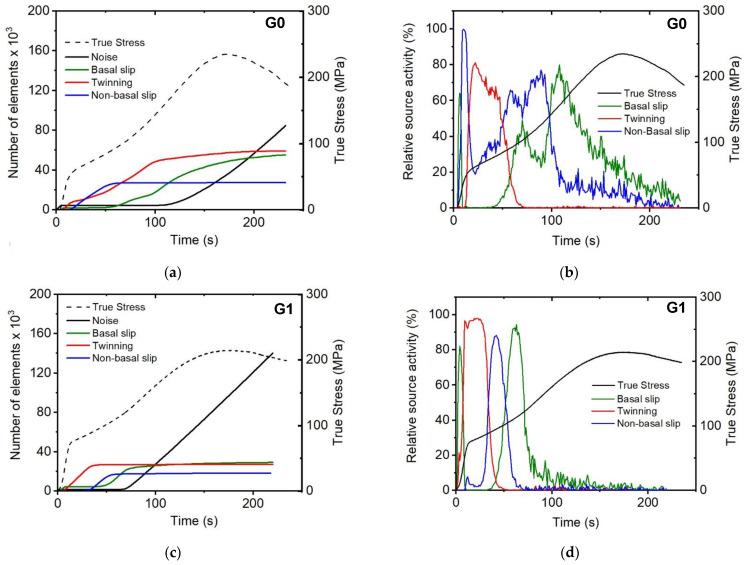

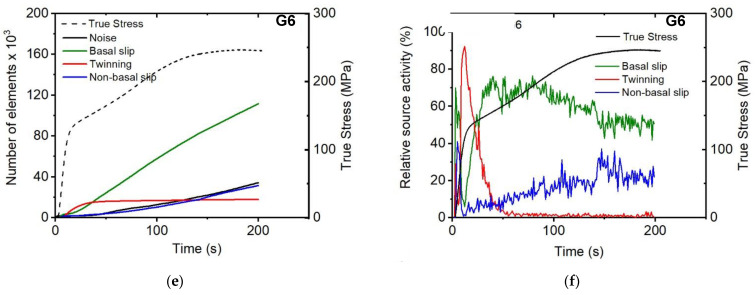
Time evolution of the cumulative number of elements in the AE clusters assigned to noise (black line), basal slip (olive line), twinning (red line), and non-basal slip (blue line) for (**a**) G0; (**c**) G1, and (**e**) G6 samples. The dashed lines represent the experimental stress strain curves, measured concurrently with AE. The relative AE source activities calculated from (**a**,**c**,**e**) for (**b**) G0, (**d**) G1, and (**f**) G6 samples. The color code of the clusters is the same as in Figure 8 and Figure 9, the black lines represent the deformation curves.

Four clusters were found by ASK, which are plotted in the energy–median frequency representation for sample G1 as an example in Figure 8 (the data for other two samples look similar). The noise cluster (Figure 8a) is characterized by a low energy and wide frequency spectrum that appear before the beginning of the compression test [62]. The other three clusters (Figure 8b–d) have been associated with basal slip, tensile twinning, and dislocation slip in non-basal planes, respectively. In Figure 9a–c, the time evolutions of the elements in the particular cluster are plotted for all samples.

Basal slip: The events associated with this cluster already appear at low applied stress, before yield stress (Figure 9a–c). The cluster has a characteristic teardrop shape (Figure 8b) in the low and medium energy range, indicating its dislocation origin [63]. Since the value of the resolved stress on the basal slip shear plane is very small [64], as is the observance of the microyielding in the in situ diffraction experiments, the assignment of this group to this mechanism is plausible.

Tensile twinning: The cluster associated with twinning appears at the beginning of plastic deformation (Figure 9a–c). The energy of the signals is high compared with the other two mechanisms (Figure 8c). Twinning has a small critical shear resolved stress [64], and numerous authors [57,59,65,66] have described the yielding in the textures of magnesium alloys under compression caused by twinning. Low-frequency signals appear exclusively at the beginning of deformation and are connected to twin nucleation in grains with larger grain size, where twinning begins earlier [59] and where the twin propagation pathway is longer than in smaller equiaxed grains. This leads to a wider signal range.

Non-basal slip**:** As is obvious from Figure 8d, the cluster associated with the non-basal also has a teardrop shape, similar to the basal slip cluster. The appearance of this cluster is in the plastic zone, which is in good agreement with the observations of other authors (e.g., [57,58]), who have discovered the significant role of the non-basal slip activity in the plasticity of magnesium alloys.

The relative activity of each source, plotted in Figure 9b,d,f, can be calculated as a time derivative of Figure 9a,c,e. The compression curve obtained during the in situ test has also been represented in order to compare the activity of each deformation system with the different regions of the compressive curve. It is obvious that at the beginning of the plastic deformation of the three materials the slip of basal <a>-dislocations is the dominant mechanism. The appearance of this cluster can be observed virtually from the onset of straining. This result is in excellent agreement with the microyielding observed in the in situ diffraction results in grains oriented with the {10$\bar{1}$1} planes perpendicular to the compression axis. Then, still in the elastic regime, the twin nucleation activity increases continuously. This process controls the plastic deformation up to the strain values of 4–5%. It is important to point out that the AE signal is sensitive exclusively to the twin nucleation and propagation (i.e., its longitudinal growth) [63]. The twin thickening (lateral growth) is undetectable by AE [63]. Therefore, it is comprehensible that above 3% of compressive strain, the twinning activity tends to disappear. Finally, the signal of the non-basal systems has been observed after the disappearance of twin nucleation. Although this signal disappears quickly in favor of the basal system for the extruded pure magnesium and the extruded G1 alloy. In the G6 alloy, the activation of non-basal slip systems has been continuously observed up to high compressive plastic strain.

### 3.5. Microstructure of Deformed Samples

**Figure 10:**
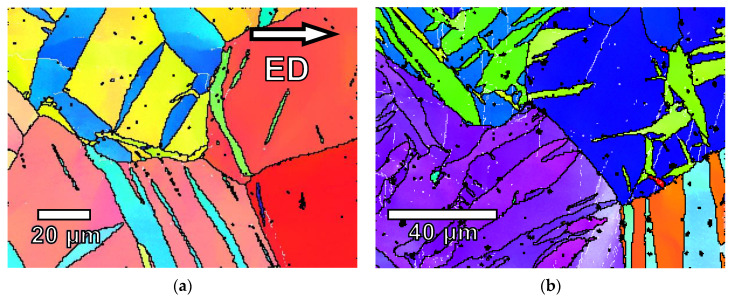

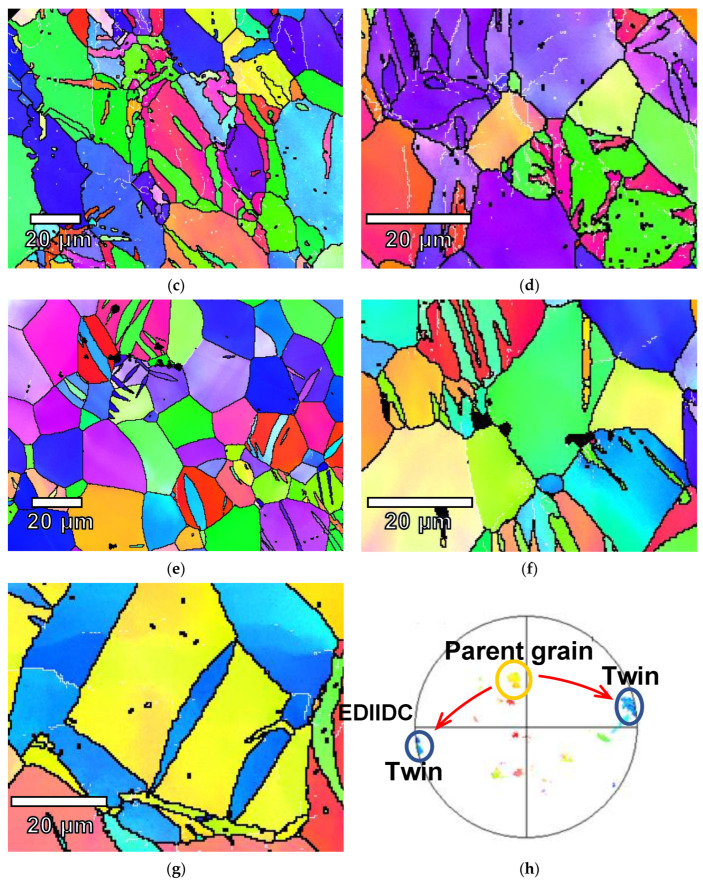
Orientation image mappings of extruded (**a**,**b**) G0, (**c**,**d**) G1, and (**e**,**f**) G6 alloys and along the extrusion direction deformed up to (**a**,**c**,**e**) 2% and (**b**,**d**,**f**) 6% of plastic compressive strain. (**g**) Detailed image of the OIM of (**a**,**h**) (0002) pole figure corresponding to (**g**) showing the lattice rotation of the lattice within twins with respect to the parent grain.

Figure 10a–f shows the orientation image map (OIM) obtained from the samples tested under compression at different compression strains. Figure 10a,b shows the orientation map images for the extruded pure magnesium (G0) deformed up to a compressive strain of 2% and 6%. At 2% plastic strain, the microstructure shows a high density of twins homogeneously distributed within the magnesium grains. The activation of the twin system {10$\bar{1}$2}〈10$\bar{1}$1〉 induces a rotation of the crystal lattice within the 86° twin. Figure 10g shows a feature of the twins generated during the deformation and the (0002) pole figure corresponding to the image. Twins (blue color) are rotated from the original grain (yellow color) which exhibits a crystallographic texture with the basal plane parallel to the extrusion direction (Figure 10h). Within the parent grain and twins, it is possible to distinguish different shades of color. The magnesium lattice is slightly rotated both in the magnesium matrix and inside the twins. This rotation, with respect to the initial orientation, can be seen in the pole figure, where the intensity is extended to nearby orientations. This effect could be produced by the activation of an additional slip systems within the twins, which induces a slight rotation of the crystal lattice compared to the deformation induced by tensile twinning.

As the compressive deformation progresses, twin boundaries grow laterally along the original grain until they completely change their initial orientation. At 6% of compressive plastic strain, the grains are almost completely twinned.

Figure 10c–f shows the microstructure of the extruded alloys G1 and G6 during plastic deformation at room temperature where the compression test was stopped at 2 and 8% of plastic strain. Although it is difficult to estimate the difference in twin volume fraction between the different materials, it appears that the proportion of twins decreases as the gadolinium content increases. This result agrees with the data obtained from the (0002) pole figures (Figure 6e). Furthermore, the twin thickness also decreases with the increase in gadolinium.

**Figure 11:**
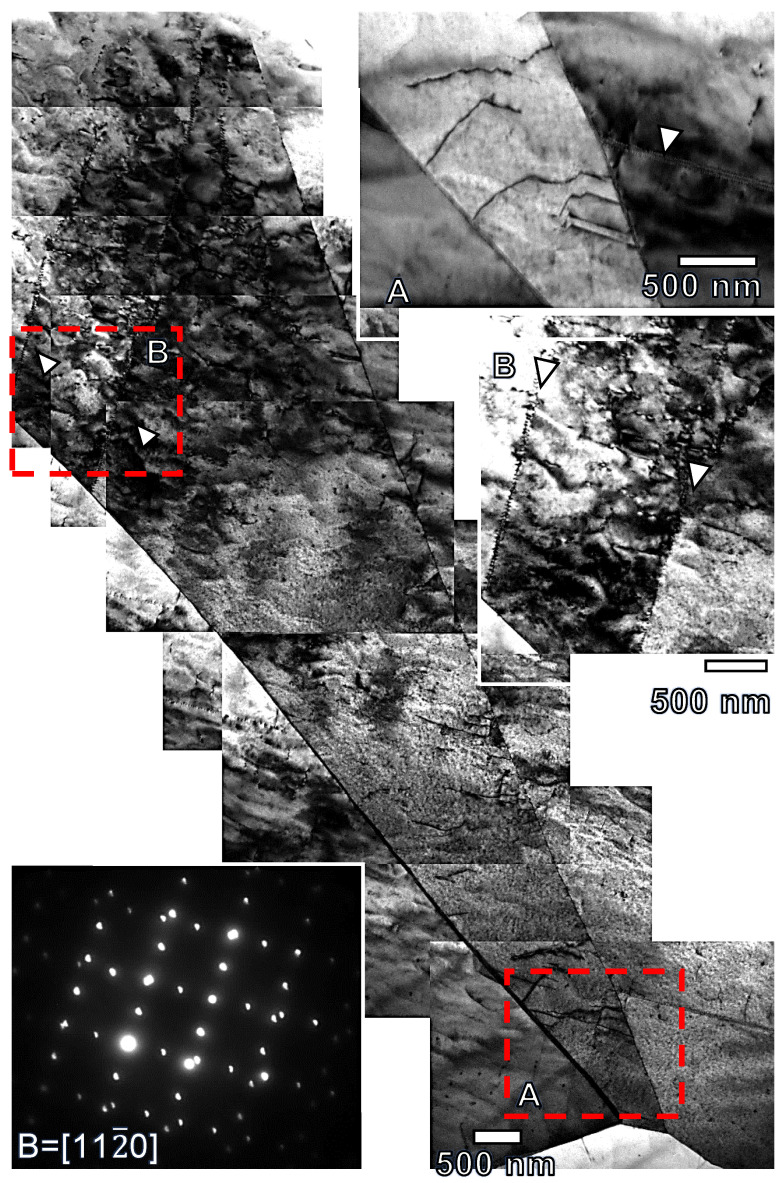
Bright field TEM image of the extruded G0 alloy compressed up to 2% of plastic deformation at room temperature (zone axis B = [11$\bar{2}$0]) and details of the twin showing dislocation arrays within the twin and the parent grain (white arrows). A and B areas in the central figure (red rectangles) are enlarged in the A and B figures. SADP of the magnesium grain and the twin (zone axis B = [11$\bar{2}$0]m,t).

Figure 11 shows two weak beam TEM images of the extruded pure magnesium sample (G0) at 2% of plastic compressive strain. Selected area electron diffraction (SAED) of zone axis B = [11$\bar{2}$0] shows the lattice rotation (around 86°) between the magnesium parent grain and the twin. Within twins, dislocations and dislocation arrays are observed. These dislocation arrays generate the disorientation observed within the twins in Figure 10g.

**Figure 12:**
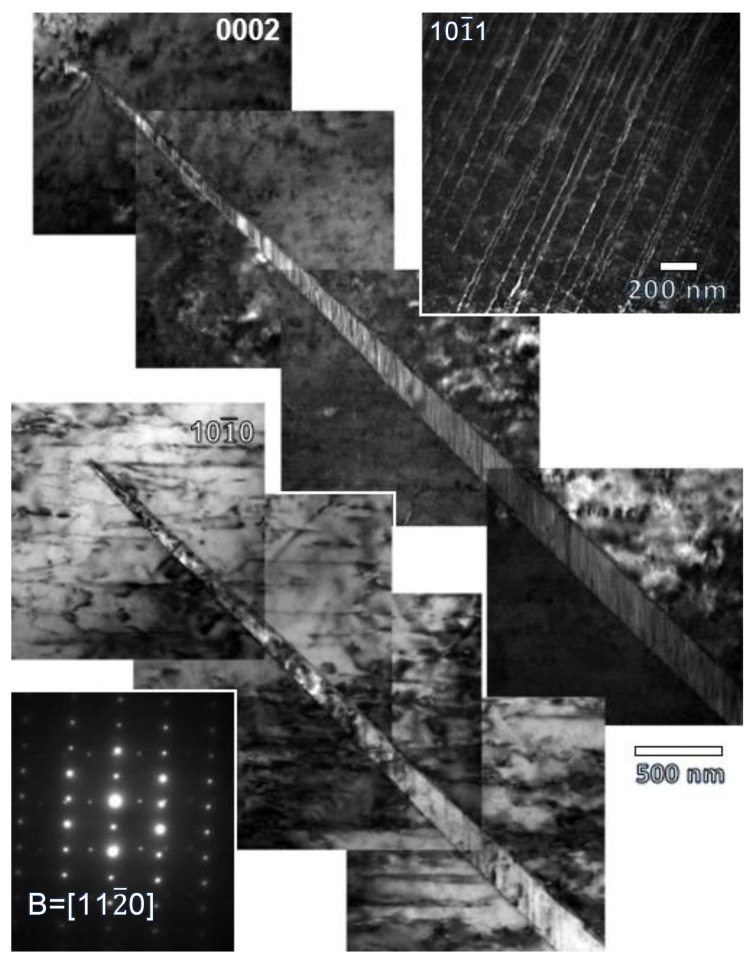
Weak beam TEM image of the extruded G6 alloy compressed up to 2% of plastic deformation at room temperature: zone axis B = [11$\bar{2}$0]_m,t_ and g = [0002]_m_, g = [10$\bar{1}$0]_m_ and g = [10$\bar{1}$1]_m_. SADP of the magnesium grain and the twin (zone axis B = [11$\bar{2}$0]m,t).

Figure 12 shows two beam TEM images of the extruded magnesium at 2% of plastic compressive strain. Both TEM images are obtained in the same zone axis B = [11$\bar{2}$0]. However, the images have been obtained by exciting different diffraction vectors g, 0002 and 10$\bar{1}$0, respectively. A tensile twin inside the grain can be observed. Selected area electron diffraction of zone axis B = [11$\bar{2}$0] shows the lattice rotation (around 86°) between the magnesium parent grain and the twin (Figure 12). Given the epitaxy relationship between the original magnesium grain and the twin, in the zone axis B = [11$\bar{2}$0], the diffraction vector g = (10$\bar{1}$0)_m_ in the matrix is the same diffraction vector g = (0002)_t_ in the twin, and the diffraction vector g = (0002)_m_ of the matrix is the same diffraction vector g = (10$\bar{1}$0)_t_ of the matrix. The subscripts m and t refer to the matrix and the twin, respectively.

In the original grain (Figure 12), the presence of dislocations parallel to the basal plane is observed, which are visible for the diffraction vector g = (10$\bar{1}$0)_m_ and invisible for the diffraction vector g = (0002)_m_. This implies that the Burgers vector of these dislocations is 1/3<11$\bar{2}$0>. A detail of these dislocations can be seen also in the weak beam image in Figure 12. Additionally, parallel and straight fringes are observed within the twin when the diffraction vector g = (10$\bar{1}$0)_t_ in the twin is excited (g = (0002)_m_ in the original grain) (Figure 12). This is characteristic of stacking faults generated by Shockley dislocations. The stacking faults are parallel to the basal plane and generate diffuse lines along the [0002] direction in the electron diagram of the twin.

**Figure 13:**
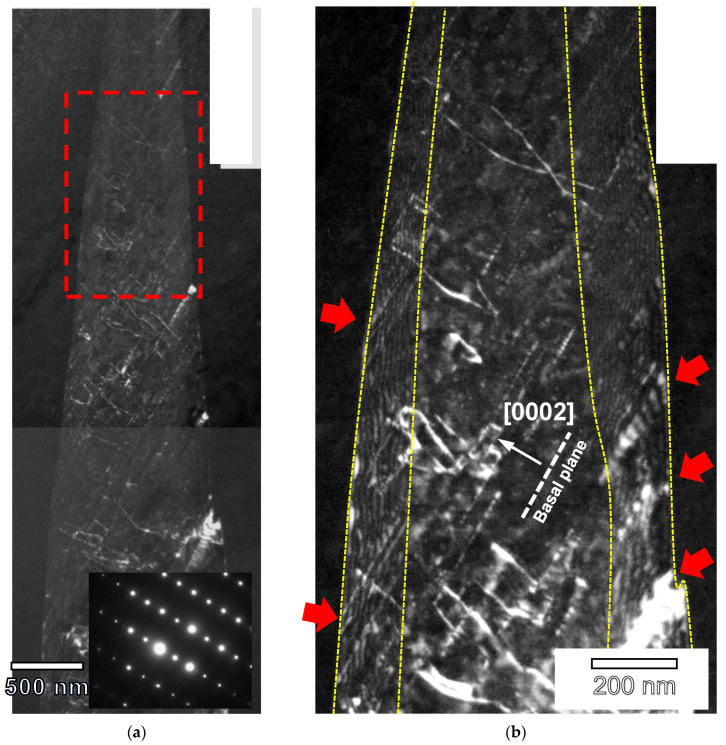
(**a**) Weak beam TEM image of the extruded G6 alloy compressed up to 2% of plastic deformation at room temperature showing the dislocation structure within twins (zone axis B = [11$\bar{2}$0]_m,t_, g = [0002]_t_). (**b**) Detail of (**a**) corresponding to the red square. Red arrows corresponds to steps in the twin boundary.

In Figure 13, dislocations are also observed inside the twins. The dislocations are visible for the diffraction vector g = (0002)_t_ and invisible for g = (10$\bar{1}$0)_t_. Therefore, the dislocations observed inside the grain are of the <c> and/or <c + a> type.

Dislocations are also observed along the twin boundary. These dislocations would be the twin-type dislocations (b_t_ = 0.063 [10$\bar{1}$1]) that form the twin boundary. Furthermore, it is possible to observe dislocations perpendicular to the previous ones that interact strongly with them (red arrows) forming steps in the twin. These dislocations seem to be related to the dislocations that form outside the twin corresponding to the basal system and that would remain on the boundary during the transmutation process [67].

## 4. Discussion

### 4.1. Influence of Gadolinium Solute Atoms on the Yield Stress

The addition of gadolinium to magnesium alloys has had a great effect on grain refinement, obtaining values of 42 ± 1 and 23 ± 1 µm for extruded G1 and G6 alloys, respectively. This refinement agrees with the previous research on Mg-Gd and Mg-Y binary alloys [68,69,70]. This grain refinement induces an increase in mechanical strength, as observed in Figure 2a. The quenching process carried out during the extrusion induces the solid solution gadolinium atoms in the magnesium matrix. Different mechanisms have been proposed to explain the increase in the mechanical strength of Mg-Gd binary alloys. As is well known, the solid solution effect and grain refinement are the main contribution [28,30,70,71]. Xu et al. [30] have demonstrated that the fundamental strengthening mechanism is the solid solution hardening.

The contribution of solid solution to mechanical hardening increases as the gadolinium content increases, while the rest of other contributions remain almost constant [30]. This contribution is given by the following expression [30]:

(4) $$∆\sigma =Bc^{n}$$

where B is a constant that depends on the elastic modulus of magnesium and the different size between the atoms of the matrix and the solute and *n* is the exponent of the concentration of solute atoms, c, with a value of 1/2 or 2/3 [72,73]. The higher the solute content or the value of B, the higher the effect of solid solution in hindering the onset of plastic deformation. Gao and et al. [28] have described that gadolinium atoms increase the bond energy between gadolinium and magnesium atoms and even between magnesium atoms. Stanford et al. [68] have reported that the addition of gadolinium in solid solution produces an increase in the yield stress, which agrees with the results of this study. However, these authors comment that the effect is especially significant up to a content value of 1% (wt.%). These authors have also shown that the greatest influence of the gadolinium solute atoms is on the prismatic deformation system.

Yang et al. [42] have proposed that the yield strength in binaries Mg-Gd alloys can be calculated throughout the different reinforcement contributions,

(5) $$\sigma_{0.2}=\sigma_{0}+∆\sigma_{HP}+∆\sigma_{ss}$$

where σ_0_ is the friction stress, σ_ss_ is the solid solution contribution, and σ_HP_ is the grain size contribution. The grain size contribution is given by the Hall–Petch equation:

(6) $$∆\sigma_{HP}=\frac{k}{\sqrt{D}}$$

where k is a constant of each material and D is the grain size. Kula et al. [41] have obtained the k value for the binaries Mg-Gd alloys (k_Gd_ = 158 MPa µm^1/2^). In order obtain the σ_ss_ contribution, the Labusch [72] theory has been used:

(7) $${∆\sigma }_{s}=B_{Gd}c_{Gd}^{2/3}$$

The value of the B_Gd_ constant has been obtained by Toda-Caraballo et al. [74]. The calculated values obtained from Equation (5) of 58 and 102 MPa are obtained for the G1 and G3 alloys, respectively. These values differ from the experimental values by about 20 MPa. Alloys studied in this work, opposite to [42], have a stronger texture and coarser grain size. Therefore, this difference could be related to the effect of texture. Grains oriented with the basal planes parallel to the extrusion direction induces the activation of the tensile twinning, which controls the yielding. The presence of gadolinium in solid solution could increase the CRSS of the tensile twinning much more than for the slip systems. An eightfold increase is expected in the tensile twinning CRSS because of the gadolinium addition [75]. Gadolinium atoms raise the apparent twin nucleation stress around the 29% with respect to pure Mg [76]. However, the atomic size mismatch theory for solid solution strengthening cannot sufficiently explain the strength increment observed in some magnesium alloys [28,77,78]. The formation of short-range order clusters has been reported in Mg-Gd alloys which effectively interact with dislocations [64].

### 4.2. Influence of Gadolinium Solute Atoms on Twinning Activity and Work Hardening

Both in situ experiments (SRD and AE) coincide in assigning the yielding of the materials under compression with the activation of tensile twinning. In addition to twinning, both in situ experiments have shown evidence of the activation of the basal and non-basal slip system at different stages of the compression curves. Already in the macroscopic elastic regime the grains oriented with their {10$\bar{1}$1} planes perpendicular to the compression axis undergo plastic deformation by basal slip.

In the vicinity of the yield point, the signal corresponding to the twin nucleation becomes dominant in the AE spectrum (Figure 9a). The apparent discrepancy between the continuous increase in intensity of the (0002) diffraction peak, indicating a continuous increase in twin volume, and the disappearance of the twin-related AE cluster in the secondary hardening zone can be explained as follows: whereas the diffraction tests characterize the volume fraction of twins, the AE signal is sensitive only to the twin nucleation and propagation, it is “proportional” to the number of the twins. Since the twin growth (increment in volume), which takes place particularly in the later stage of deformation, is almost six orders slower of a process than the twin propagation [79], it is undetectable by the AE technique.

It is expected that gadolinium atoms in solid solution have an influence on the three consecutive stages of twinning: nucleation, propagation, and growth.

Tang et al. [80] has claimed that presence of solute tends to decrease the nucleation energy of twins in the case of Mg-(Al, Zn, or Y) alloys. Although, this behavior would change for higher solute contents that tend to suppress twin nucleation above a threshold solute content in Mg-Al or Mg-Y binary alloys [80,81]. It is expected that gadolinium atoms hinder twin nucleation similarly to yttrium atoms. For a Mg-5%Gd single crystals, alloy the twin nucleation stress has been estimated as 152 MPa, which is 34 MPa higher than that for pure magnesium.

The propagation stage is the fastest stage compared to nucleation or lateral growth. The driving force for the propagation stage is the reduction in the stress generated at the tip of the twin. It is expected that solute atoms interact with twin dislocations of the twin boundary, although the calculated tension at the tip of the twin would be high enough to minimize this effect [82] and solute atoms would have a minimal influence in hindering or delaying the propagation of twins from one side of the grain to the opposite one. However, experimental results have recently shown that gadolinium atoms in solid solution slow down the twin propagation rate [79] since the twin tip moves intermittently due to the presence of the solute atoms.

Finally, solute atoms also increase the stress required for the lateral growth of the twin, since it increases the Peierls stress. Ghazisaeidi et al. [83] have calculated the effect of the presence of solute for the lateral growth of a twin in the AZ31 alloy from first principles, obtaining a value of about 14 MPa. Although this value is not large, it is higher than the effect of the solute interaction for the slip of <a> dislocations along the basal plane. It should be expected that gadolinium atoms have stronger behavior than aluminum or zinc atoms due to their larger atom size. Therefore, the increase in the nucleation stress as well as in the stress for lateral growth explains the decrease in the twinning activity and the decrease in the twin thickness with the increasing gadolinium content. These effects directly influence the evolution of the work hardening (Figure 2b).

**Figure 14:**
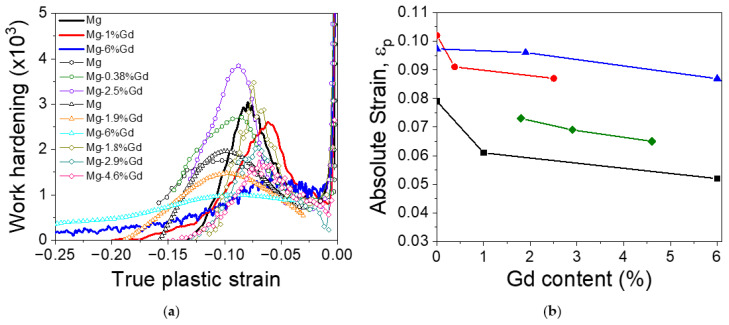
(**a**) Comparison of the evolution of work hardening as a function of the compressive strains of this study (continuous lines) and [41] (diamonds), [42] (triangles) and [43] (circles). (**b**) Evolution of the strain of the hardening peak of region II of (**a**). (black square correspond to data obtained in this study, red circles correspond to data obtained in [43], blue triangles correspond to data obtained in [42] and green diamonds correspond to data obtained in [41]).

Figure 14a compares the evolution of the work hardening exponent obtained in this work with values reported in the literature [41,42,43]. After the initial minimum, the work hardening increases again with the increase in the compressive strain up to reach a peak due to the formation of twins. As a general behavior, the compressive strain corresponding to the maximum of the peak, ε_p_, is shifted to lower strain values with the increasing gadolinium content (Figure 14b). However, values for ε_p_ found by other researchers exhibit a large scatter, which indicates a much more profound dependence of this parameter on microstructural features. In general, the evolution of the ε_p_ with gadolinium content is similar in our study and in Refs. [41,42] (cf. Figure 15), where the microstructures have consisted of fully equiaxed recrystallized grains. In contrast, a weak influence of the Gd content on the ε_p_, was reported by Imandoust et al. [43] for a bimodal of structure, having a high-volume fraction of coarse and elongated grains oriented with the basal plane parallel to the extrusion direction. In such a structure, twinning preferentially occurs in these coarse grains at much lower stresses than in the fine recrystallized grains [59].

The hardening peak in the compression tests stems from the increase in the dislocation density within twins and the interaction between twins in the same grain [45,84]. Ma et al. [85] suggested that at least the upper half of the hardening regime in the compression curve should be characterized by the dominance of the dislocation slip within twins. The dislocation density within grains increases rapidly due to the transmutation process of dislocations from the parent grains towards twins through the twin boundaries. The first step of the transmutation process corresponds to the transformation of a <a>_m_ dislocation into a twin dislocation b_t_, that are retained into the twin boundary, and a <c + a>_t_ dislocation [67] (indices m and t mean matrix and twin, respectively). The b_t_ dislocation induces a step into the twin boundaries that are observed in Figure 13b. In the second step, the <c + a>_t_ dislocation is dissociated into:

(8) $$\frac{1}{3}\langle \bar{1}2\bar{1}3\rangle \to \langle 0001\rangle +\frac{1}{3}\langle \bar{1}2\bar{1}0\rangle \to \langle 0001\rangle +\frac{1}{3}\langle 01\bar{1}0\rangle +\frac{1}{3}\langle \bar{1}100\rangle$$

where the 〈10$\bar{1}$0〉 Shockley dislocations lying in the basal plane are responsible for the staking faults observed within twins (Figure 12). The other dislocations observed in Figure 14 within the twin correspond to <c> dislocations coming from the transmutation process or <c + a> coming from the activation of non-basal deformation systems. Inmandoust et al. [43] proposed that RE favors the transmutation process. Therefore, at the same plastic strain, the dislocation density can be higher if gadolinium in solid solution is added. Therefore, the work hardening is higher and ε_p_ is shifted towards a lower plastic strain in comparison to pure magnesium. However, binary Mg-Gd alloys with recrystallised equiaxed grains contradict this initial assumption. The solid solution of gadolinium, in addition to increasing the stress for the nucleation and growth of twins, induces the decrease in the intensity of the texture fiber with the basal plane parallel to the extrusion direction. Then, there are less grains favorably oriented for the activation of twinning, and the volume fraction of twin decreases with the gadolinium addition (Figure 6e). Therefore, even when transmutation was favored by the presence of gadolinium in solid solution, their influence on the work hardening is less intense than for pure magnesium.

## 5. Conclusions

The combination of in situ synchrotron diffraction and acoustic emission experiments during compression tests at room temperature of Mg-Gd alloys has been used to analyze the effect of gadolinium in solid solution on the plastic deformation of magnesium. The following conclusions can be drawn:

(1) The main strengthening mechanism of binary Mg-Gd alloys at room temperature turned out to be the solid solution of the solute atoms in the magnesium matrix. Evidently, this contribution increases with the increasing gadolinium concentration. The difference between the experimental and theoretical values is due to the possible formation of gadolinium short-range order clusters.
(2) The tensile twinning system {10$\bar{1}$2}〈10$\bar{1}$1〉 is the deformation mechanism dominant during the plastic deformation under compression along the extrusion direction above the macroscopic yield. The presence of gadolinium atoms in solid solution tends to hinder this mechanism, particularly the lateral growth of twins. Therefore, the volume fraction of twins at the same plastic compressive strain decreases with the increasing gadolinium content.
(3) In addition to twinning, the activation of the basal and pyramidal slip systems has been observed in binary Mg-Gd alloys. The slip of <a> dislocations along the basal plane is activated before reaching the yield point. The activation stress for this microyielding increases with increasing gadolinium content. The addition of gadolinium also favors the activation of non-basal slip system at higher applied stresses.
(4) The work hardening curve in the extruded alloys presents a peak at the intermediate plastic strain between 0.04 and 0.09 depending on the concentration of gadolinium. This peak is related mainly to the interaction of dislocations within twins. The dislocations density inside the twins increases rapidly with the amount of gadolinium in solid solution as a consequence of the transmutation of<a>-type dislocations across twin boundaries from the parent grains. Nevertheless, since the volume fraction of twin decreases with the increasing gadolinium content, the hardening value of the hardening peak in region II decreases.

## Data Availability

Data are contained within the article.
